# Blink and You'll Miss It: Diagnostic Pitfalls in a Case of Missed Ocular Myasthenia Gravis in the Emergency Department

**DOI:** 10.7759/cureus.90976

**Published:** 2025-08-25

**Authors:** Joshua Neumann, Mohammad S Aqil, Simran P Singh, Dema Fawaz

**Affiliations:** 1 Medical School, Oakland University William Beaumont School of Medicine, Rochester, USA; 2 Emergency Medicine, Corewell Health Beaumont Troy Hospital, Troy, USA

**Keywords:** acetylcholinesterase inhibitors, diagnostic delay, emergency department, fatigable ptosis, ice pack test, ocular myasthenia gravis

## Abstract

Ocular myasthenia gravis (MG), a subtype of MG limited to the eyelids and extraocular muscles, is diagnostically challenging in the emergency department (ED). In the absence of generalized weakness, subtle symptoms such as ptosis and diplopia are more easily overlooked or misattributed to stroke or other neurologic pathologies. We present a case of a 69-year-old male whose diagnosis of ocular MG was delayed due to limited neurologic examination, underuse of simple bedside tools, and incidental imaging findings that initially suggested alternative endocrine pathology. Despite classic signs, including fatigable ptosis and diplopia, treatment with pyridostigmine was not initiated until 31 hours after ED arrival, at which point the patient experienced near-complete resolution of ocular symptoms within one hour. This case highlights the importance of maintaining clinical suspicion for MG in cranial nerve presentations. Incorporating accessible bedside tests, such as the ice pack test or acetylcholinesterase inhibitor trial, can expedite diagnosis and treatment in the ED.

## Introduction

Myasthenia gravis (MG) is the most common disorder of neuromuscular transmission, yet it can pose a diagnostic challenge, particularly in older patients with only ocular manifestations [1,2]. Ocular MG, a subset of MG in which symptoms are restricted to the eyelids and extraocular muscles, can present with fluctuating ptosis and diplopia, features that can mimic microvascular cranial nerve palsies or stroke [1,2]. These subtle symptoms may be difficult to recognize in the emergency department (ED) setting, where diagnostic accuracy is challenged by limited time, reliance on rapid decision-making, and variable access to specialist evaluation. Simple bedside tests, such as applying an ice pack to the eyelid or a short trial of an acetylcholinesterase inhibitor, can provide rapid clinical support for suspected ocular MG and are easily implemented in the ED [2].

Particularly, the diagnosis of ocular MG can be missed or delayed in the ED due to underrecognition of its hallmark clinical features, overreliance on imaging, and underuse of simple bedside tests. This issue is not unique to the ED, as diagnostic delays in MG are common, with average time to diagnosis exceeding one year; ocular-predominant cases are often delayed by over 14 months [3]. Such delays can lead to prolonged patient evaluation, unnecessary testing, delayed treatment initiation, and increased risk of progression from ocular to generalized MG [2]. 

We present a case of ocular MG in an older adult whose evaluation in the ED was prolonged by several diagnostic challenges, including incidental imaging findings that complicated clinical reasoning and delayed therapeutic trials. This case underscores the importance of maintaining clinical suspicion and incorporating bedside assessments to improve diagnostic efficiency in emergency care. 

## Case presentation

Emergency department evaluation

This patient is a 69-year-old Caucasian man with a history of hypertension and hyperlipidemia who initially presented to a free-standing ED in Southeast Michigan with complaints of bilateral drooping eyelids and double vision. He reported a three-month history of blurred vision, followed by progressive right eyelid ptosis beginning approximately six weeks prior and left eyelid ptosis starting the day before presentation to the ED. There were no other neurologic symptoms, including weakness, numbness, breathing problems, or dysphagia. The patient denied alcohol use and exposure to botulinum toxin. The patient had a 20-pack-year smoking history but had quit 20 years prior. There was no family history of neuromuscular or thyroid disorders. 

At presentation, a non-contrast head computed tomography (CT) scan was obtained; findings were unremarkable and thus not included. He was then transferred to our hospital-based ED in Southeast Michigan for further workup and evaluation. Upon arrival, initial workup included a CT angiogram and venogram (CTA/V) of the head, which were unremarkable for acute intracranial or vascular pathology. However, imaging incidentally revealed a 2.3 x 1.4cm thyroid nodule within the right thyroid lobe. Although the CTA/V images were not available for inclusion, the incidental thyroid nodule prompted further workup. Thyroid function tests, including thyroid-stimulating hormone (TSH), free T3, and free T4, were within normal limits. Other laboratory findings were unremarkable and are summarized below (Table 1). 

**Table 1:** Relevant lab results from emergency department WBC, white blood cell count; RBC, red blood cell count; Hgb, hemoglobin; Hct, hematocrit; MCV, mean corpuscular volume; BUN, blood urea nitrogen; AST, aspartate aminotransferase; ALT, alanine aminotransferase; TSH, thyroid stimulating hormone

Test	Value	Reference Range
WBC	7.5	3.5-10.1 x 10^9^/L
RBC	4.61	4.31-5.48 x 10^12^/L
Hgb	13.3	13.5-17.0 g/dL
Hct	44.1	40.1-50.1%
MCV	98.6	79.5-100.4 fL
Platelet Count	232	150-400 x 10^9^/L
Creatinine	0.96	0.60-1.30 mg/dL
BUN	26	7-25 mg/dL
AST	27	<35 U/L
ALT	25	9-47 U/L
TSH	3.09	0.40-4.50 mclU/mL
Free T3	2.3	1.7-3.7 pg/mL
Free T4	0.7	0.7-1.5 ng/dL

Physical examination in the ED noted bilateral ptosis and extraocular movement abnormalities. Specifically, there was impaired lateral gaze bilaterally, with medial gaze weakness in the right eye. Pupils were equal and reactive. There was no documentation of fatigue testing or specific neuromuscular signs such as the curtain sign. No other abnormalities were noted on physical examination, including cardiovascular, respiratory, abdominal, skin, or neurological systems. Bedside diagnostic testing, such as the ice pack test or an acetylcholinesterase inhibitor trial, was not performed in the ED. The ED team focused on ruling out acute cerebrovascular events, including ischemic stroke and vascular cranial neuropathies. Given the thyroid nodule, suspicion was raised for thyroid ophthalmopathy. The patient was admitted to the hospital for neurology, ophthalmology, and endocrine consultations.

Inpatient management and follow-up

**Figure 1:**
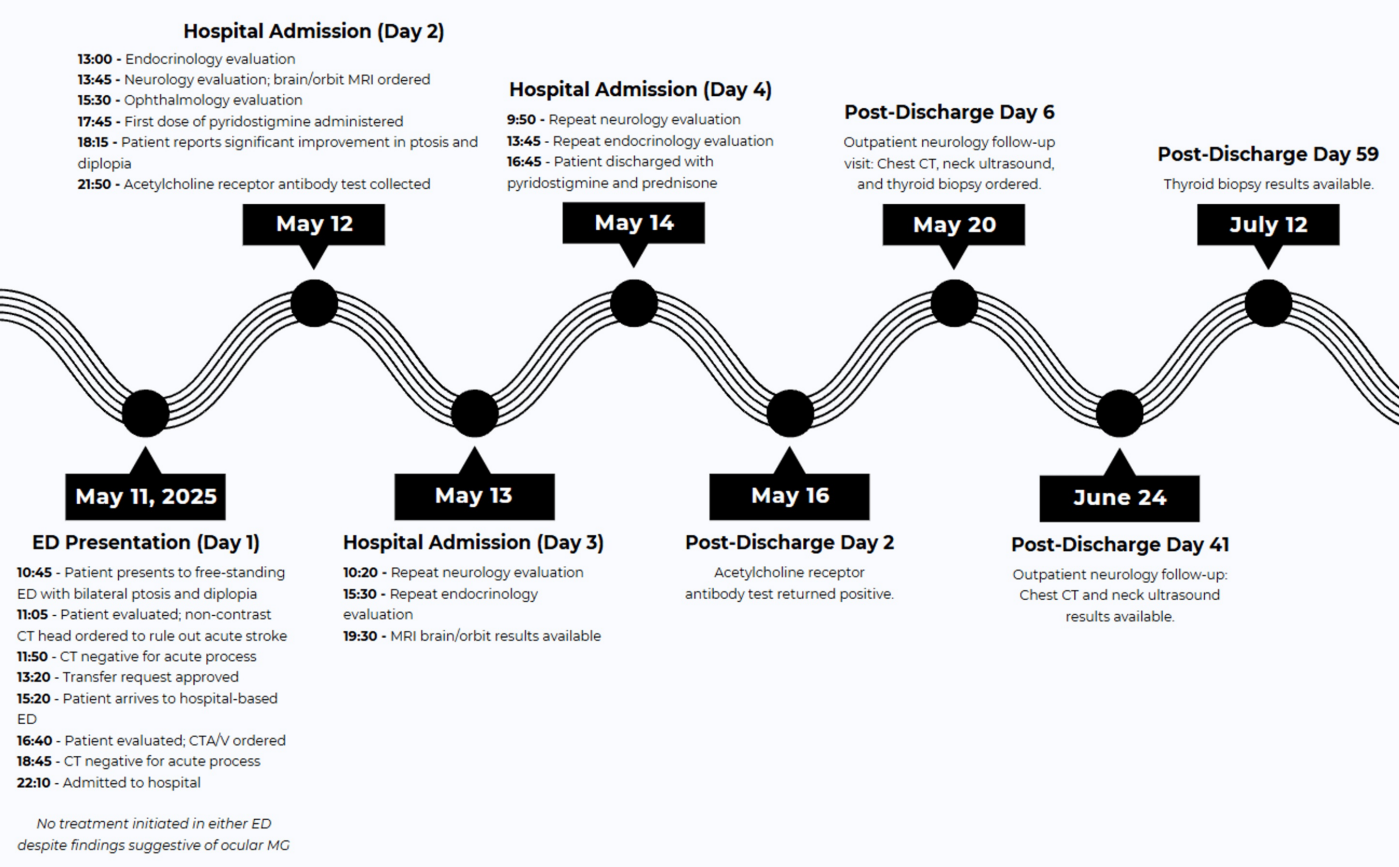
Timeline of major clinical events from emergency department presentation through post-discharge follow-up in a 69-year-old male with ocular myasthenia gravis ED, emergency department; CT, computed tomography; CTA/V, computed tomography angiography/venography; MRI, magnetic resonance imaging

The neurology service first assessed the patient approximately 27 hours after initial ED arrival, on hospital day 2. This delay was likely because the patient was admitted late in the evening and the case was considered non-urgent, consistent with standard overnight staffing limitations. Neurology evaluation revealed severe bilateral ptosis, limited extraocular movements, and a positive curtain sign. The neurology service strongly suspected ocular MG and recommended initiation of pyridostigmine bromide. Ophthalmology assessment confirmed binocular diplopia, bilateral ptosis with fatigability, limited extraocular movements, and nuclear sclerotic cataracts; pyridostigmine was similarly recommended. Pyridostigmine was started approximately 31 hours after initially presenting to the ED, with marked improvement in eyelid droop and diplopia within just 30 minutes of administration. Earlier bedside testing or neurology evaluation might have shortened the diagnostic delay. The timeline of events is illustrated in Figure 1.

Endocrinology evaluated the patient’s thyroid nodule, with plans for outpatient thyroid ultrasound and biopsy. Additional thyroid tests, including thyroid-stimulating immunoglobulin and thyrotropin receptor antibody, were normal. Acetylcholine receptor antibody testing, sent during admission, later returned positive two days post-discharge (Table 2).

**Table 2:** Relevant lab results from admission

Test	Value	Reference Range
Thyroid stimulating immunoglobulin	<0.10	<0.10 IU/L
Thyrotropin receptor antibody	<1.10	0.00-1.75 IU/L
Acetylcholine receptor modulating antibody	99	<=45%

**Figure 2:**
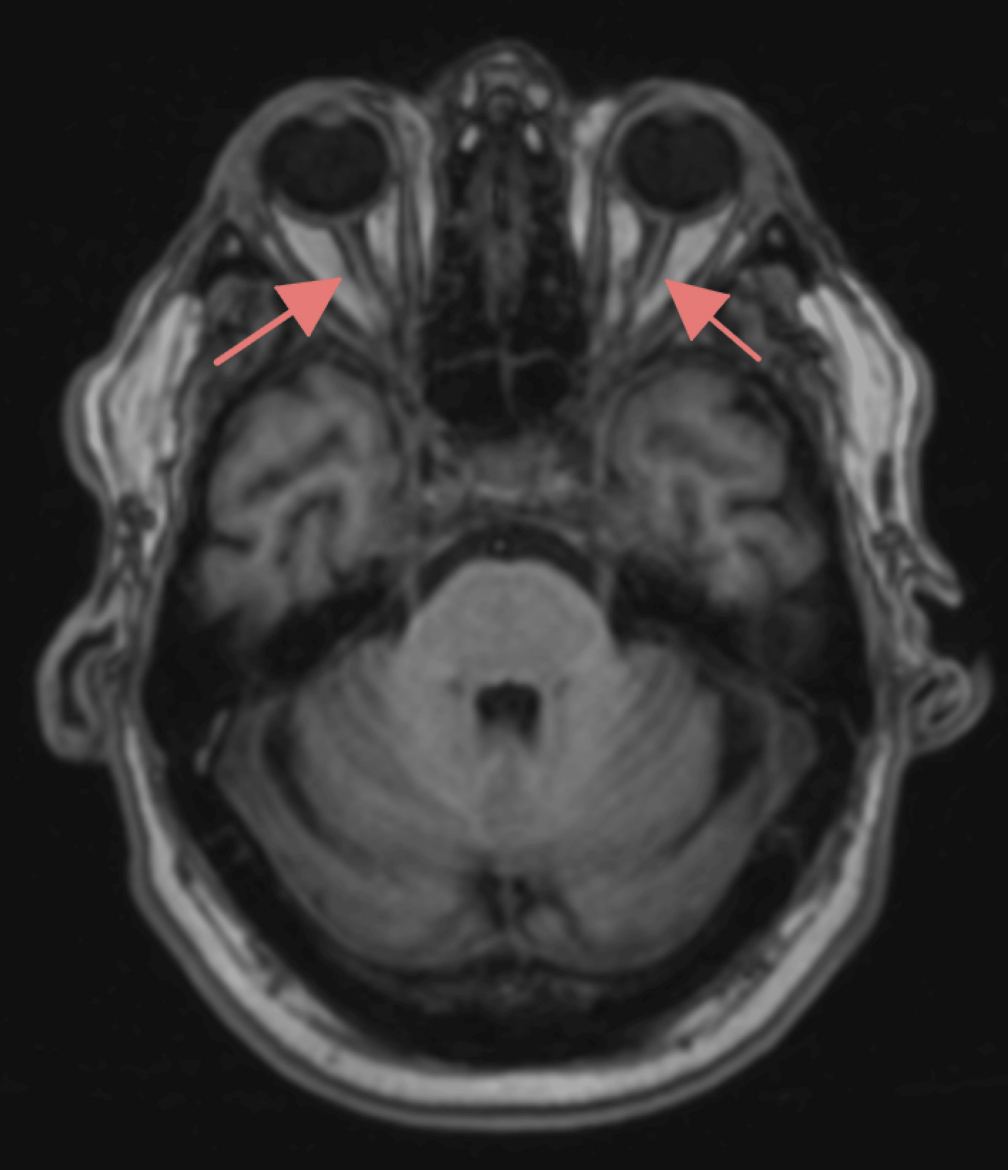
Axial T1-weighted MRI of the orbits Bilateral optic nerves appeared somewhat atrophied with diminutive caliber (red arrows). There is no enlargement of the extraocular muscles, arguing against thyroid ophthalmopathy. No acute ischemia or mass effect. MRI, magnetic resonance imaging

Magnetic resonance imaging (MRI) of the brain and orbits was obtained on hospital day 2, with results available on hospital day 3, demonstrating no acute ischemia or mass effect (Figure 2). There was no enlargement of the extraocular muscles, arguing against thyroid ophthalmopathy. Bilateral optic nerves appeared somewhat atrophied with diminutive caliber. These largely unremarkable MRI findings, combined with clinical improvement on pyridostigmine, supported a working diagnosis of ocular MG. The patient was discharged on hospital day 4 with instructions to continue pyridostigmine and oral prednisone. 

**Figure 3:**
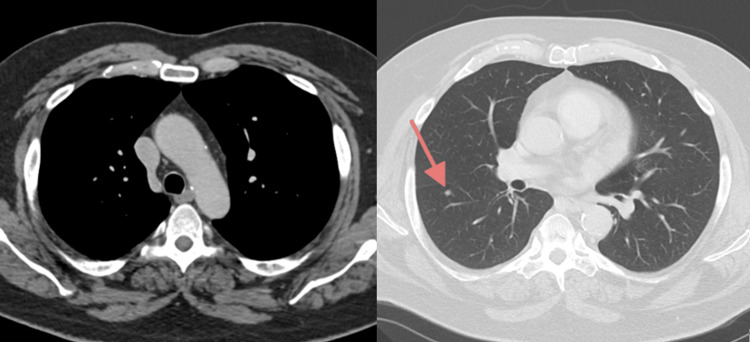
Axial chest CT images in soft tissue window (left) and lung window (right) There is no evidence of mediastinal mass or thymoma. An incidental 5mm pulmonary nodule noted in the right lower lung lobe (right image, arrow), for which surveillance imaging was recommended. CT, computed tomography

At outpatient neurology follow-up six days after discharge, azathioprine was initiated as additional immunosuppressive therapy. CT scan of the chest obtained as an outpatient ruled out thymoma but revealed a small right lower lobe nodule, warranting surveillance imaging (Figure 3). Thyroid ultrasound confirmed the right thyroid nodule, with ultrasound-guided fine needle aspiration of the right thyroid nodule demonstrating benign follicular cells consistent with a colloid nodule. There was no evidence of malignancy or suspicious cytologic features. Due to limited clinical relevance and image availability, these additional imaging studies were not included. Two months post-discharge, the patient reported significant symptomatic improvement on his medical regimen. 

## Discussion

This case highlights several diagnostic pitfalls that delayed recognition of ocular MG in the ED. One of the most significant pitfalls in this case was the overreliance on imaging and attribution of symptoms to incidental findings. The patient’s CT angiogram incidentally revealed bilateral proptosis and a right thyroid nodule, findings that shifted the working diagnosis towards thyroid eye disease despite normal thyroid function tests and no clinical or family history of thyroid dysfunction. This represents a form of anchoring bias, the tendency to prematurely anchor on a finding that appears to explain the presentation [4]. In reality, the radiographic findings were incidental, and the patient lacked other features of thyroid eye disease, such as lid retraction, pain, or muscle enlargement. Overemphasis on these findings delayed consideration of ocular MG and unnecessarily broadened the diagnostic workup. 

A second pitfall was the incomplete neurologic examination in the ED. The patient’s presentation included fluctuating ptosis and diplopia, and although limited extraocular movements were documented, more detailed assessments were omitted. The curtain sign, worsening of one eyelid’s ptosis when the contralateral lid is manually elevated, was not tested until neurology’s evaluation on hospital day two. This sign reflects impaired neuromuscular transmission of the levator palpebrae muscles and is a validated clinical indicator in ocular MG [5]. Its absence in the initial ED exam highlights how incomplete assessments, whether due to time pressure, documentation gaps, or limited familiarity with neuromuscular diagnoses, can delay key clinical clues that would otherwise guide early diagnosis. 

The third and perhaps most actionable pitfall was the underuse of simple bedside diagnostic tools, specifically the ice pack test and a trial of acetylcholinesterase inhibitors. Historically, the edrophonium (Tensilon) test was used to support MG diagnosis by transiently improving ptosis following intravenous administration [5]. Due to potential cardiovascular risks and the availability of safer, non-invasive bedside tests like the ice pack test, its use is no longer routinely recommended in the ED [5]. The ice pack test takes advantage of the fact that cooling improves neuromuscular transmission in MG by inhibiting acetylcholinesterase activity [6]. Studies report a sensitivity of approximately 80-90% and a very high specificity for ocular MG, though it should not be considered completely definitive [6]. Applying an ice pack to the affected eyelid for two to five minutes often results in transient improvement in ptosis and can provide rapid clinical support for an ocular MG diagnosis, especially in the ED [6,7].

Similarly, pyridostigmine is an oral acetylcholinesterase inhibitor that enhances neuromuscular transmission by increasing acetylcholine availability at the motor endplate [8]. In this patient, a single dose produced marked improvement within 30 minutes, effectively confirming the diagnosis before anti-acetylcholine receptor antibody testing resulted. This outcome is consistent with existing evidence indicating that acetylcholinesterase inhibitors can produce prompt, low-risk symptom relief in patients with MG [8]. Early implementation of either test in the ED could have expedited both diagnosis and therapeutic intervention.

Together, these pitfalls illustrate a broader theme: a lack of a structured approach to isolated cranial nerve presentations in the ED, where non-emergent but time-sensitive conditions like ocular MG can fall through the cracks. When life-threatening causes such as aneurysm or stroke must be excluded, providers should remain cautious of cognitive closure after normal imaging and consider neuromuscular causes when findings are fatigable, symmetrical, and fluctuating.

Earlier bedside testing, more complete neurologic examination, and cautious interpretation of incidental findings may have led to more timely diagnosis and treatment. Emergency physicians should maintain a broad differential for isolated cranial nerve symptoms and utilize accessible bedside maneuvers when appropriate. Without the use of simple bedside tools such as the ice pack test or a low-risk trial of acetylcholinesterase inhibitors, some patients with ocular MG may remain undiagnosed for several months or even years, as documented in prior studies reporting average diagnostic delays exceeding one year for this subtype [3,9]. Implementing institutional checklists, such as the International Consensus Guidance for the Management of Myasthenia Gravis, may help reduce variability in the assessment of neuromuscular presentations and further improve recognition of less common, but highly treatable, conditions like ocular MG [10].

## Conclusions

Ocular MG is a diagnostically challenging condition in the emergency setting due to its subtle presentation and overlap with more acute neurologic diseases. This case illustrates how incidental imaging findings and incomplete bedside assessments can lead to delays in appropriate diagnosis and treatment. Simple bedside tools such as the ice pack test and a trial of pyridostigmine are underused despite their high diagnostic utility. Greater awareness of these pitfalls and strong encouragement for the use of bedside assessments can help emergency physicians recognize ocular MG earlier and initiate timely, targeted therapy. 
